# Recent Updates on Molecular and Physical Therapies for Organ Fibrosis

**DOI:** 10.3390/molecules30244766

**Published:** 2025-12-13

**Authors:** Michał Filipski, Natalia Libergal, Maksymilian Mikołajczyk, Daria Sznajderowicz, Vitalij Novickij, Augustinas Želvys, Paulina Malakauskaitė, Olga Michel, Julita Kulbacka, Anna Choromańska

**Affiliations:** 1 Faculty of Medicine, Wroclaw Medical University, Mikulicza-Radeckiego 5, 50-345 Wroclaw, Poland; michal.filipski@student.umw.edu.pl (M.F.); natalia.libergal@student.umw.edu.pl (N.L.); maksymilian.mikolajczyk@student.umw.edu.pl (M.M.); daria.sznajderowicz@student.umw.edu.pl (D.S.); 2 Student Research Group No. SKN148, Faculty of Pharmacy, Wroclaw Medical University, Borowska 211A, 50-556 Wroclaw, Poland; 3 Department of Molecular and Cellular Biology, Faculty of Pharmacy, Wroclaw Medical University, Borowska 211 A, 50-556 Wroclaw, Poland; vitalij.novickij@imcentras.lt (V.N.); augustinas.zelvys@imcentras.lt (A.Ž.); paulina.malakauskaite@imcentras.lt (P.M.); olga.michel@umw.edu.pl (O.M.); anna.choromanska@umw.edu.pl (A.C.); 4 Department of Immunology and Bioelectrochemistry, State Research Institute Centre for Innovative Medicine, Santariskiu g. 5, LT-08406 Vilnius, Lithuania; 5 Faculty of Electronics, Vilnius Gediminas Technical University, Plytinės g. 27, LT-10105 Vilnius, Lithuania

**Keywords:** antifibrotic therapy, fibrosis, gene therapy, stem cells, targeted therapy

## Abstract

Organ fibrosis is a progressive and often irreversible pathological process characterized by excessive deposition of extracellular matrix, leading to tissue dysfunction and failure. Despite its significant impact on various organ systems, available antifibrotic therapies remain limited. This review focuses on novel therapeutic approaches to inhibit fibrosis and improve clinical outcomes. Current strategies include small molecule inhibitors, monoclonal antibodies targeting fibrosis mediators, gene therapies, and cell-based approaches, including mesenchymal stem cells and induced pluripotent stem cells. In addition, the development of innovative drug delivery systems and combination therapies involving pulsed magnetic fields (PMFs) opens new possibilities for increasing the precision and efficacy of treatment. In recent years, multiomic approaches have enabled a better understanding of fibrosis mechanisms, facilitating the personalization of therapy. The role of artificial intelligence in drug discovery has also increased, as exemplified by models that support the design of small-molecule inhibitors currently undergoing clinical evaluation. This review discusses key signaling pathways involved in fibrosis progression, such as TGF-β, p38 MAPK, and fibroblast activation, as well as novel therapeutic targets. Although clinical trial results indicate promising potential for new therapies, challenges remain in optimizing drug delivery, considering patient heterogeneity, and ensuring long-term safety. The future of fibrosis therapy relies on integrating precision medicine, combination therapies, and molecularly targeted strategies to inhibit or even reverse the fibrosis process. Further intensive interdisciplinary collaboration is required to successfully implement these innovative solutions in clinical practice.

## 1. Introduction

Maintenance of a proper wound healing response is crucial for the efficient functioning of the organs of the body [1]. Deregulation of the process leads to excessive deposition of extracellular matrix (ECM) components, such as collagen and fibronectin, resulting in the formation of scar tissue in all organs and, subsequently, their dysfunction and failure [2,3]. Chronic inflammatory diseases, acute or chronic ischemia, hypertension, long-lasting viral infection (viral hepatitis), environmental exposure (smoking, alcohol, pneumoconiosis), and genetic diseases (cystic fibrosis, alpha-1-antitrypsin deficiency) are among the main causes of organ fibrosis [1,3]. It is a highly progressive and irreversible disease that is classified as a leading cause of morbidity and mortality worldwide [2,4]. Moreover, there are also cases detected in children [5]. Additionally, fibrosis significantly impacts treatment and the development of malignant tumors, affecting carcinogenesis, invasiveness, and metastasis, as well as drug delivery to masses [2]. Due to the lack of effective anti-fibrotic therapies, it is important to investigate new targets for innovative treatment. In clinical trials targeting organ fibrosis, modulating inflammatory and immune molecules, or regulating fibroblast function and ECM deposition are emerging therapeutic aims. An innovative approach is focused on the development of monoclonal antibodies against fibrotic mediators, gene therapies, and even stem cell transplantation. To design effective anti-fibrotic therapeutics, an integrated genetic, biological, and biophysical concept will be needed. Moreover, it is believed that to succeed in finding a treatment, the perception of organ fibrosis as an inflammatory process should be abandoned [3].

## 2. Understanding the Pathophysiology of Organ Fibrosis

### 2.1. Cellular and Molecular Mechanisms Underlying Fibrosis

A key group of cells in the fibrosis process is fibroblasts and myofibroblasts. Under the influence of profibrotic cytokines, particularly TGF-β, fibroblasts become activated and differentiate into myofibroblasts, which are the main effector cells in fibrosis [3,6]. Myofibroblasts are characterized by the production of α-smooth muscle actin (α-SMA) and increased synthesis of extracellular matrix [3]. Additionally, the fibrosis process involves epithelial–mesenchymal transition. Epithelial cells lose their cell–cell adhesion properties and polarity, transitioning to a mesenchymal phenotype that exhibits greater migratory capacity and the ability to produce extracellular matrix. Similarly, endothelial cells transform into mesenchymal cells, thus increasing the pool of activated fibroblasts [3,7].

### 2.2. Role of Inflammation, Fibroblasts, and Extracellular Matrix Remodeling

Inflammation acts as a trigger in the fibrosis process. Both acute and chronic inflammatory processes induce fibrosis in many tissues of the body [8]. The inflammatory factor is believed to have a decisive influence on the course of this process, and its removal is a relatively simple way to stop the progression of tissue remodeling. Unfortunately, quickly removing the inflammatory trigger is not always possible, as it is often unknown [3]. Initially, tissue damage triggers an inflammatory response characterized by the accumulation and activation of leukocytes in the damaged tissue [9]. This leads to the release of pro-inflammatory cytokines (e.g., IL-1, IL-6) and chemokines that recruit immune cells to the injury site [3]. Macrophages adopt an anti-inflammatory M2 phenotype. If the damage is uncontrolled, M2 macrophages undergo persistent activation, leading to continuous production of TGF-β and other growth factors that promote myofibroblast proliferation [10]. T cells also participate in this process: Th2 cells release IL-4, IL-6, and IL-13, thereby further promoting myofibroblast activation and extracellular matrix production [9]. Fibroblasts are recognized as a heterogeneous population composed of distinct subtypes that arise from different developmental lines and respond differently to injury. These cells have distinct subpopulations that differ in their capacity to produce extracellular matrix components [11]. Some subsets, such as perivascular or inflammatory fibroblasts, are primed to respond to cytokines and rapidly transition into an activated state. In contrast, others, including matricellular or myofibroblast precursors, have a disproportionately high capacity to synthesize collagen and other extracellular matrix proteins. Single-cell transcriptomic studies demonstrate that these subsets exhibit unique gene expression profiles, signaling sensitivities, and functional roles during tissue remodeling, explaining why only specific fibroblast populations drive pathological matrix accumulation in fibrosis [12,13,14].

Chronic inflammation and cytokine signaling activate fibroblasts and drive their differentiation into myofibroblasts, which are the primary collagen producers in injuries [15]. Under physiological conditions, when the damaging factor and the inflammatory response subside, the extracellular matrix is resorbed, and the healing process proceeds correctly. Matrix metalloproteinases (MMPs), regulated by tissue inhibitors of metalloproteinases (TIMPs), are responsible for tissue remodeling and ECM degradation. The resulting scars consist mainly of type I and III collagen, fibronectin, and basement membrane proteins. It is important to note that myofibroblasts also express smooth muscle proteins, which retain contractile function. The contraction of these cells further disrupts the parenchyma of the fibrosed organ, exacerbating its dysfunction [8]. Chronic inflammation is a central factor in the development of organ fibrosis, acting through persistent tissue injury and immune dysregulation to activate fibroblasts and promote their differentiation into myofibroblasts, which are key effector cells in fibrotic processes [16,17,18,19,20]. Inflammatory insults, such as those caused by drugs, infections, autoimmunity, or foreign substances, lead to tissue injury, which triggers endogenous repair mechanisms, including the transformation of fibroblasts into active myofibroblasts [21,22]. This process is observed across multiple organs, including the heart, lungs, liver, kidneys, and gastrointestinal tract [16,20,21,22]. It was noted that a variety of cytokines and chemokines are released during chronic inflammation, directly contributing to fibroblast activation and the fibrotic response. The key cytokines implicated include transforming growth factor-beta (TGF-β) [17], IL-6 family [23], IL-1β, IL-13 and IL-17, IL-10 and IL-11 [17], platelet-derived growth factor (PDGF) [20], and also other factors, such as tumor necrosis factor-alpha (TNF-α), colony-stimulating factor 1 (CSF1), and chemokines such as CCL2, CXCL1, and CXCL12, which are also involved in fibroblast activation and immune cell recruitment [23]. Activated immune cells, including macrophages, neutrophils, monocytes, and lymphocytes, can release pro-inflammatory mediators, chemokines, and growth factors that further activate fibroblasts and promote their differentiation into myofibroblasts [16,18,22,24]. In turn, fibroblasts can act as inflammatory effectors by producing cytokines and chemokines that recruit and retain leukocytes, thus amplifying the inflammatory cascade and further escalating the overall inflammatory response [16,25,26,27,28]. This mutual activation sustains chronic inflammation and drives the progression of fibrosis.

While the core mechanisms of fibroblast activation via chronic inflammation and cytokine signaling are conserved across organs, there are organ-specific differences in the cellular sources of fibroblasts, the predominant cytokines involved, and the interplay with other cell types, such as endothelial cells and pericytes [16,17,20,24,29]. For example, in the intestine and rheumatoid arthritis, specific fibroblast subsets respond to IL-6 family cytokines and contribute to local immune cell recruitment and inflammation [23]. In the liver, STAT3 signaling in hepatic stellate cells is implicated in fibroblast activation during fibrosis [30]. This mechanistic approach demonstrates that chronic inflammation and cytokine signaling are directly connected to fibroblast activation and the development of organ fibrosis, with multiple feedback loops and cell types involved in perpetuating the fibrotic response [16,17,18,20,23].

### 2.3. Signaling Pathways as a Key Target

**Figure 1:**
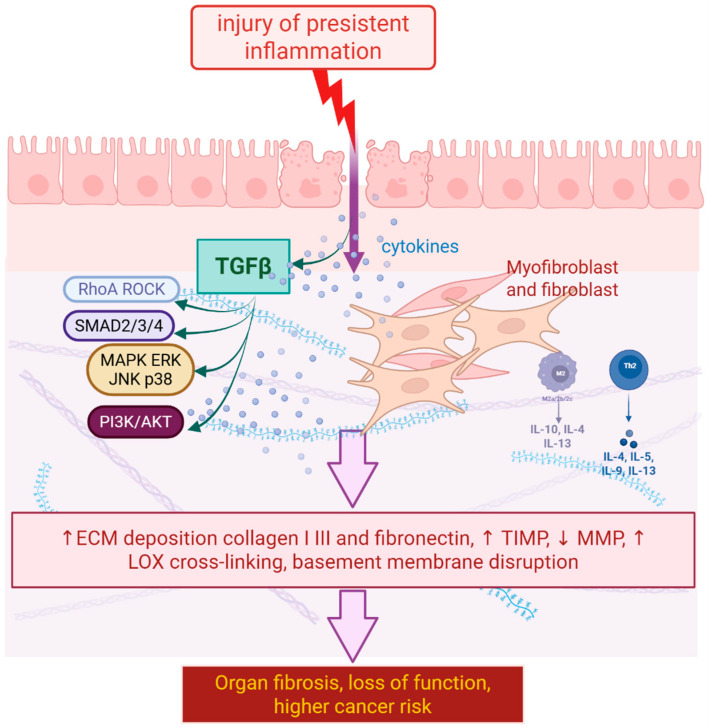
Injury or persistent inflammation triggers cytokine release and TGFβ signaling through RhoA ROCK, SMAD2 3 4, MAPK ERK JNK p38, and PI3K AKT, which activate fibroblasts and generate myofibroblasts, leading to excess ECM deposition with increased TIMP, reduced MMP, and LOX cross-linking, tissue stiffening, basement membrane disruption, organ dysfunction, and higher cancer risk (arrow up—increase, arrow down—decrease) (Created in BioRender. Kulbacka, J. (2025) https://BioRender.com/o51hkg0) [20,34].

When describing the most crucial signaling pathways involved in the fibrosis process, it is essential to start with the most important one: the transforming growth factor-beta (TGF-β) pathway (Figure 1). TGF-β binds to its type II receptor (TGF-βRII), which then recruits and phosphorylates the type I receptor (TGF-βRI). The activated TGF-βRI phosphorylates receptor-regulated Smads (R-Smads), primarily Smad2 and Smad3. Phosphorylated Smad2/3 form a complex with the common mediator Smad4, and this complex translocates to the nucleus. In the nucleus, the Smad complex regulates the transcription of target genes that promote fibrosis, such as those encoding extracellular matrix proteins (collagen, fibronectin) and tissue inhibitors of metalloproteinases [31]. After phosphorylation and nuclear translocation, receptor-regulated Smads form a complex with Smad4 that binds to Smad binding elements in target gene promoters and recruits transcriptional co-activators or co-repressors, thereby modulating chromatin structure and driving context-dependent activation or repression of profibrotic genes [32].

Another signaling pathway worth looking at is the p38 mitogen-activated protein kinase (MAPK) pathway (Figure 1). It is a key intracellular signaling pathway that contributes to the production of proinflammatory and profibrotic mediators, playing a significant role in fibrosis and extracellular matrix synthesis. When activated, p38 MAPK undergoes phosphorylation, triggering the activation of downstream kinases. There are four isoforms of p38 MAPK (α, β, γ, and δ), with α, β, and δ being the most prevalent in the kidney. Specifically, the phosphorylation of p38α leads to its movement into the nucleus, where it activates transcription factors responsible for producing proinflammatory mediators and extracellular matrix proteins [33].

### 2.4. Non-Classical Mechanisms of Fibrosis: Metabolic and Epigenetic Reprogramming

Recent research has expanded the understanding of fibrosis beyond classical pathways, highlighting the importance of metabolic reprogramming and epigenetic regulation as key non-classical mechanisms in fibrotic diseases across multiple organs, including the heart, liver, kidney, and lung [35,36,37,38,39,40]. Mechanisms are now recognized as central to the activation and behavior of fibroblasts and other cell types involved in fibrosis.

Metabolic reprogramming refers to alterations in cellular energy metabolism, notably a shift towards enhanced glycolysis, changes in lipid metabolism, and alterations in amino acid metabolism in fibroblasts and other cells during fibrosis. This reprogramming supports the increased energy and biosynthetic demands of activated fibroblasts, contributing to excessive extracellular matrix (ECM) production and perpetuation of the fibrotic process [35,36,39,40,41,42,43]. In renal fibrosis, glycolytic reprogramming initially serves an adaptive function. However, it becomes pathogenic, with lactate production and mitochondrial dysfunction driving fibrosis [36,40]. In liver fibrosis, metabolic and immune dysregulation, often triggered by hypoxia and mitochondrial dysfunction, are closely linked to fibrotic progression [44,45]. Metabolic intermediates, such as lactate, can directly influence epigenetic modifications (e.g., lactateylation), creating feedforward loops that sustain pro-fibrotic gene expression [46,47]. Metabolic reprogramming modulates signaling pathways, including TGF-β/Smad, MAPK, STAT3, and AMPK, impacting inflammatory and oxidative stress responses [21,36,39,41].

Epigenetic regulation represents a second non-classical axis that stabilizes fibrotic phenotypes at the transcriptional level. Epigenetic regulation involves DNA methylation, histone modifications (acetylation, methylation), and non-coding RNAs (ncRNAs), as well as emerging epitranscriptomic modifications such as N6-methyladenosine (m6A) [35,37,38,48,49,50,51]. These mechanisms can lock fibroblasts and macrophages into persistent profibrotic pathways even after the initial insult has resolved. This has initiated the development of small-molecule inhibitors, therapeutic miRNAs, and other molecules targeting profibrotic gene networks. Inhibitors of DNA methylation and histone-modifying enzymes (e.g., HDAC inhibitors) have shown promise in preclinical models. However, their application in clinical fibrosis remains limited. It requires improved specificity [38,52]. NcRNA-based and CRISPR-based epigenetic editing approaches are emerging as potential therapeutic tools, with challenges related to delivery, stability, and targeting [35,38,53]. Targeting m6A modifications and their interplay with DNA methylation and ncRNAs is a novel area of investigation [48]. Together, these approaches illustrate how targeting metabolic and epigenetic reprogramming may complement upstream anti-inflammatory and anti-TGFβ therapies and better show the current direction of antifibrotic drug discovery.

Metabolic reprogramming and epigenetic regulation represent critical, non-classical mechanisms in the pathogenesis of fibrosis, offering novel therapeutic targets. While significant progress has been made in understanding these pathways, translation into effective clinical therapies remains a major challenge. Currently ongoing research (Table 1) is focused on overcoming these barriers and integrating these insights into comprehensive antifibrotic strategies.

## 3. Innovative Therapeutic Approaches

### 3.1. Small Molecule Inhibitors and Targeted Therapies

To date, only two drugs—Nintedanib and Pirfenidone—have been officially approved for the treatment of idiopathic pulmonary fibrosis (IPF). Pulmonary, hepatic, renal, and cardiac fibrosis share common mechanisms, such as chronic inflammation, fibroblast activation, and ECM deposition, yet each organ exhibits distinct cellular drivers and molecular targets. These drugs are currently the two approved antifibrotic agents for idiopathic pulmonary fibrosis, but they differ in indications, efficacy profile, and tolerability, which guide clinical decision-making [55,56]. Both drugs slow the decline in forced vital capacity (FVC) to a comparable extent in phase III trials, reducing annual FVC loss by roughly 50% compared with placebo. Yet, pirfenidone has additional data suggesting a reduction in all-cause mortality in some cohorts [55]. In contrast, nintedanib has broader indications, extending to progressive fibrosing interstitial lung diseases and non-IPF in several regions. Their safety profiles also differ: nintedanib is predominantly associated with gastrointestinal adverse events, particularly diarrhea and reversible liver enzyme elevations, while pirfenidone more often causes photosensitivity, skin rash, and gastrointestinal discomfort, requiring sun protection and careful dose titration. In practice, these differences mean that age, comorbidities (for example, chronic liver disease or photosensitive skin disorders), concomitant medications, and patient preference often determine the initial choice of agent, and close monitoring of adverse events may prompt switching between nintedanib and pirfenidone or dose adjustments to maintain long-term antifibrotic therapy [57,58]. Nintedanib acts by inhibiting the differentiation of fibroblasts into myofibroblasts as well as their migration and proliferation. Pirfenidone, on the other hand, is an inhibitor of TGF-β, a key component of one of the most important fibrotic pathways. It suppresses the production and release of pro-inflammatory cytokines. Despite their proven efficacy in slowing the progression of these processes, both drugs are used primarily to decelerate the decline in lung function in patients with IPF. However, they fail to improve survival rates or significantly enhance the quality of life [59,60]. Additionally, these treatments are often associated with bothersome side effects, particularly gastrointestinal issues and skin reactions [60].

Given these limitations, there is an urgent need for innovative and effective therapies targeting the fibrotic processes underlying IPF and other organ fibrosis. In liver and renal fibrosis, current therapies mostly focus on metabolic and immune-mediated pathways [40,44,46], whereas cardiac fibrosis research is intensifying around TGFβ blockade [61,62], modulation of myofibroblast mechanotransduction, and antiremodeling strategies. Current research is investigating the safety and efficacy of combination therapies while also exploring the antifibrotic potential of drugs already well known and widely used in other indications. Among these are cardiovascular drugs, such as lisinopril, a lysine derivative of enalapril. Lisinopril, a potent inhibitor of angiotensin converting enzyme, has shown promise in reducing hydroxyproline and collagen levels in pulmonary fibrosis models by inhibiting angiotensin II and TGF-β1 [60]. Similarly, interest has emerged in certain antidiabetic drugs, particularly metformin. Preclinical studies have shown that metformin can reduce lung inflammation and fibrosis at both early and late stages of the disease. Its antifibrotic effects are achieved through multiple mechanisms, including activation of AMPK pathways, inhibition of TGF-β signaling, reduction in collagen and fibronectin production, deactivation of myofibroblasts, and suppression of macrophage and fibroblast activation [60]. These examples illustrate both the convergence of fundamental profibrotic cascades and the need for specific organ therapeutic approaches that account for unique microenvironmental and biomechanical constraints. While research into these repurposed drugs remains ongoing, the most promising therapeutic targets are the new small-molecule inhibitors

### 3.2. Biologics and Gene Therapies

Knowledge of the specific cells and cytokines involved in the organ fibrosis enables scientists to invent treatments targeting them. The most focused methods involve using monoclonal antibodies against fibrotic mediators and gene-editing techniques to modulate fibrotic gene expression. Therapy based on monoclonal antibodies has been largely investigated over the last decade, and many drugs are still in clinical trials. However, some of them, such as Pamrevlumab and BG00011, have been shown to be effective and safe in IPF patients. Pamrevlumab is a human antibody against CTGF that is being investigated in a phase 3 RCT. Anti-αvβ6 integrin monoclonal antibody, BG00011, is being conducted in a phase 2a randomized, placebo-controlled trial. Moreover, investigators, after proposing that autoantibodies may be involved in IPF, conducted a trial demonstrating improvements in gas exchange with TPE (therapeutic plasma exchange) and rituximab. Additionally, patients had a 1-year survival rate of 46%, whereas none were alive at 1 year in the historical control group [63,64]. Other therapeutic antibodies under investigation for IPF include monoclonal antibodies to IL-13 (e.g., lebrikizumab), which have been shown to be effective in poorly controlled asthma and to reduce fibrosis in animal models [3,4]. Clinical trials have also been initiated for therapeutic antibodies to TGF-β1, which activate myofibroblasts, and for humanized monoclonal antibodies targeting lysyl oxidase-like-2 (which catalyzes collagen cross-linking) as therapies for cardiac fibrosis, IPF, and liver fibrosis. A phase 1 study of a human monoclonal antibody targeting CCL2, which recruits inflammatory monocytes, is also underway. Researchers provided clinical proof-of-concept for specific monoclonal antibodies targeting CLDN1 for the treatment of advanced liver, kidney, and lung fibrosis. Therefore, this indicates CLDN1 as a therapeutic target for organ fibrosis [65].

Given that gene therapy is an innovative tool, few are registered for treating organ fibrosis. However, scientists have introduced some promising concepts. One of them is a targeted AAV9-Tspyl2 gene therapy designed to restore cell division autoantigen-1 (CDA1) expression in various organs and tissues. This therapy targets the Tspyl2 gene located on the X chromosome. CDA 1, as a transcription regulator, inhibits the profibrotic effects of TGF-β1 without side effects in murine models of atherosclerosis and renal fibrosis. In addition, these studies show that Tspyl2 gene therapy inhibits the lung fibroblast-to-myofibroblast transition and downstream TGF-β/Smad3 signaling transduction in BLM-induced PF in mice. All things considered, AAV9-Tspyl2 gene therapy is a promising therapy [66]. Another futuristic therapy targets effector cells in liver fibrosis. The main subject of the study is the CRISPR/dCas9 system, which enables site-specific transcriptional regulation and is highly efficient at activating gene transcription for robust outcomes [67]. In the case of pulmonary fibrosis caused by Hermansky–Pudlak syndrome (HPS1), gene therapy is being used to deliver a fully functional gene using DNA, RNA, or oligonucleotides, using viral or non-viral vectors, to restore the function of a specific protein-encoding gene [68].

### 3.3. Cell-Based Therapies

Cell-based therapies for organ fibrosis, particularly those involving induced pluripotent stem cells (iPSCs), offer promising avenues for treatment (Table 2). iPSCs can self-renew and differentiate into various cell types, making them a valuable resource in regenerative medicine. In the context of organ fibrosis, iPSCs can be differentiated into specific cell types relevant to the affected organ, such as macrophages, alveolar epithelial cells, hepatocytes, cardiomyocytes, and endothelial cells. These differentiated cells can then be used to replace damaged or dysfunctional cells, thereby reducing fibrosis and promoting tissue repair [69]. For example, iPSC-derived macrophages can reduce fibrogenic gene expression and histological markers in liver fibrosis models. In contrast, iPSC-derived alveolar epithelial cells can re-epithelialize injured alveoli, restoring pulmonary function and reducing lung fibrosis [70,71,72]. In myocardial fibrosis, iPSC-derived cardiomyocytes can reduce fibrosis and improve cardiac function [69]. Beyond direct cell replacement, iPSCs can also be used to produce extracellular vesicles and exosomes, which mediate cell-to-cell communication and have shown potential to reduce fibrosis-promoting markers and activate anti-fibrotic pathways [73]. Conditioned media from iPSCs can also increase alveolar epithelial wound repair and attenuate fibrosis by blocking the TGF-β/Smad pathway [74]. Table 2 below summarizes novel therapeutic possibilities that are approved or on the research level.

## 4. Preclinical and Clinical Studies

### 4.1. In Vitro and Animal Model Studies Evaluating Efficacy and Safety

For now, there are two drugs approved for the treatment of idiopathic pulmonary fibrosis: Nintedanib and Pirfenidone. Unfortunately, neither is the perfect choice because of their limited therapeutic potential. In more advanced cases, the solution is either taking more drugs or adding a second one, depending on which one the patient was already taking. This can lead to side effects such as gastric and liver toxicity, and more. Lv Q et al. evaluated the efficiency of Pirfenidone [76]. This drug inhibits the secretion of profibrotic and proinflammatory factors. Moreover, it reduces inflammatory cell stimulation and postpones fibroblast proliferation and collagen deposition. Researchers conducted an in vivo test on human fetal lung fibroblasts (HLFs) and an in vitro test on mice, both cell types with bleomycin-induced fibrosis. Afterwards, they divided mouse cells and HLFs into groups treated with different substances. Our interest was only in the pirfenidone-treated groups compared with the untreated (control) groups. Results showed that pirfenidone treatment in mouse cells significantly improved alveolar structure and reduced ECM deposition. PFD treatment also caused a noticeable decrease in TNF-α and IL-6. Thanks to the therapy, HLF cells showed reduced activation. It also reduced the expression of collagen I and III mRNA.

Subsequent studies by Yang et al. reported the effects of gene modifications on MS (mesenchymal stem) cells in mice and rats with induced hepatic fibrosis [77]. MS cells were harvested from bone marrow and adipose tissue, and afterwards modified to increase gene expression. Results have shown that mice with CCL4-induced liver fibrosis and modifications of specific genes like HNF4α, FOXAa2, FGF4, SMAD7 (all four were modified in MSCs from bone marrow), and SERPINA1 (from adipose tissue) revealed significant improvements, such as promoting the expression of NF-κb signaling and enhancing the anti-inflammatory and immune regulatory effects of MSCs (HNF4α). Additionally, FOXA2 promoted hepatic differentiation of MSCs, alleviating fibrosis effects. Similar effects were observed in cells with FGF4 gene modifications, in which hepatocyte proliferation was enhanced by increased expression of PCNA, EpCAM, and Jagged1. SMAD7 gene modification resulted in MSCs with reduced levels of collagen types I and III and reduced α-SMA, TIMP1, and hyaluronic acid, which are crucial fibrosis factors, as reported in other studies. The SERPINA1 modification led to more effective differentiation of MSCs into hepatic cells. As we can observe, genetically modified MSCs can modulate the immune response and promote the proliferation of other cells. By changing the expression of many factors, they increase the therapeutic effect. It makes MSCs’ gene modification therapy very promising. However, it will take some time to start clinical trials using this method and even more time to apply it in everyday treatment.

In another study, Felix et al. tested the efficiency of AD-MSCs (adipose tissue MSCs) and their conditioned medium (CM) in the treatment of bleomycin-induced pulmonary fibrosis [78]. Rats with induced fibrosis were divided into three groups: placebo, MSCs, and CM-treated. Results showed significant improvement in fibrinogen and Von Willebrand factor levels, as well as increased expression of NOS-2 (nitric oxide synthase), indicating an anti-inflammatory effect of the treatment. TGF-β levels have gotten lower. Additionally, the conditioned medium reduced collagen-5 exposure and IL-17 levels more than the AD-MSCs group. To highlight, both AD-MSCs and their CM exerted a quality anti-inflammatory effect on pulmonary tissue. The differences were noticeable but not significant, so using AD-MSCs could also be a suitable therapy strategy in the future. Thus, both methods showed promising results for the future of pulmonary fibrosis therapy. The development of this type of treatment requires many more clinical trials and unification (for now, numerous cell types are being considered).

### 4.2. Phase I–III Clinical Trials Assessing Novel Therapies in Patients

Berh et al. conducted a study involving 127 patients [79]. They were from 18 to 80 years old and suffering from idiopathic pulmonary fibrosis for different reasons: collagen diseases, vascular diseases (ILD associated with connective tissue), fibrotic non-specific interstitial pneumonia, chronic hypersensitivity, pneumonia, and lung fibrosis induced by asbestos. Patients who were previously taking antifibrotic drugs were excluded. Participants had to have an adequate FVC and a decline in FVC of at least 5% using conventional therapies within 2 years prior to the study. They were treated with Pirfenidone at three different doses (267 mg three times a day in week 1, 535 mg three times a day in week 2, and 801 mg three times a day for the rest of the study). The placebo group was also added. The study was a double-blind, 48-week trial, and at the end, FVC improvement was measured. Unfortunately, there was a death in the group treated with PFD due to reasons unrelated to the respiratory system. 5 deaths were recorded in the placebo group, and 3 were related to the respiratory system. Adverse events, such as infections and heart problems, also occurred, all in the placebo group. The results showed the effectiveness of PFD, which significantly slowed down fibrosis. Patients improved their FVC by 3.53% (excluding deaths) and 2.79% (including). As we can see, the increase is noteworthy but not spectacular. The study not only confirmed that this drug is clinically effective but also emphasized the need for better, more targeted treatment methods.

Voelker et al. performed a randomized, double-blind phase II trial [80]. For the treatment, they used a humanized neutralizing monoclonal antibody against TGF-β (TGF-β1 mAb). The sample size was 416 patients with type 1 or type 2 diabetes. Patients have received an antibody or a placebo monthly for a year. The study did not report any safety issues during treatment, and the ratio of adverse side effects, such as end-stage kidney disease, acute kidney injury, or even death, was similar between the two groups. Surprisingly, this study did not prove the effectiveness of the antibody therapy. Another study examined a different antibody, fresolimumab, which targets all three isoforms of TGF-β (1, 2, and 3) [81]. This study also did not report significant improvements related to the treatment. However, this method requires further diagnosis to enable more targeted therapy in the future, so scientists are optimistic about it. In the study by Behrangi et al., women aged 18 to 60 with striae alba on their abdomen were divided into two groups. One of them received micro-needling (the most efficient and safe method, also used as an aesthetic therapy at the time) with normal saline (control group) [82]. The other one received micro-needling with a conditioned medium. The study was double-blind and randomized. The results showed significant improvement in thickness and density in both the epidermis and dermis. However, improvement was only slightly more visible in the group that received a conditioned medium. Even more interesting is the satisfactory level of treatment between patients and physicians—the results showed a significant improvement in satisfaction in both groups.

Another review [83] described the current state of mesenchymal stem cell therapy for liver diseases and its potential for further research. A couple of them stood out. In the first study by Suk KT et al., participating patients were struggling with alcoholic fibrosis [84]. They were treated using Autologous BM-MSCs (bone marrow). The research group consisted of 37 patients, and 18 formed the control group. The treatment lasted 6 months, and at the end, there was a significant improvement in histologic fibrosis and overall liver function. The only issue they noticed was that the exact mechanism was not elucidated. Another presented study showed treatment of HCV-related ESDL (end-stage liver disease) and HBV-decompensated cirrhosis [85,86]. The first group was treated with autologous BM-MSCs, and the second with UC-MSCs (umbilical cord). Both showed improvement in liver function and were rated as promising treatment approaches for further research. The crucial discourse of this paper was the safety of MSC treatment. The primary concern with this therapy is the risk of tumorigenesis. However, numerous studies have shown that this treatment is safe, with little to no risk of tumorigenesis years after treatment. Some studies reported side effects, mainly fever and rash, but the patient recovered without additional treatment [83]. A summary of clinical studies is shown in Table 3 below.

## 5. Biophysical and Nanomaterials-Based Antifibrotic Therapies

Besides small-molecule antifibrotics like pirfenidone and nintedanib, a new class of interventions is using precision nanomedicine and biophysics to deliver gene silencers to sentinel stromal cells, modify profibrotic signaling, and even eliminate activated fibroblasts. The ability to remotely regulate forces and drug release within tissues with millimetric accuracy makes magnetic field-based techniques and magnetic nanocarriers stand out among the others [87]. Compared with ionizing radiation, magnetic fields have considerably fewer safety restrictions and can penetrate tissue without the attenuation that light does. There are three beneficial antifibrotic strategies: (i) targeted delivery to profibrotic cell types, (ii) on-demand release or hyperthermia to reprogram signaling, and (iii) magnetomechanical disruption of pathological stromal compartments, which are made possible by the ability of superparamagnetic iron oxide nanoparticles (SPIONs) to be guided, heated, or mechanically actuated by external fields. These benefits are highlighted in reviews of liver disease, which also present SPIONs as therapeutic effectors and contrast agents (also known as “theranostics”) [88].

### 5.1. Magnetic Fields as an Antifibrotic Factor

**Figure 2:**
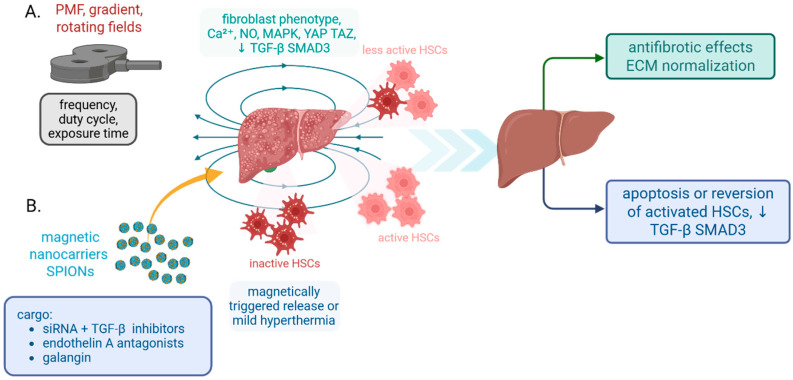
Magnetic field therapies and magnetic nanocarriers that modulate liver fibrosis. (**A**) Exposure to device-generated magnetic fields, including pulsed electromagnetic fields PEMF, pulsed magnetic fields PMF, gradient fields, and rotating fields, alters fibroblast phenotype and signaling, Ca^2+^, nitric oxide, MAPK, YAP, and TAZ, and reduces TGF beta SMAD3. This decreases hepatic stellate cell activation and promotes normalization of the extracellular matrix. Key parameters include frequency, duty cycle, and exposure time. (**B**) Superparamagnetic iron oxide nanocarriers SPIONs targeted to hepatic stellate cells deliver antifibrotic cargo such as siRNA with TGF beta pathway inhibitors, endothelin A receptor antagonists, or galangin, and can enable magnetically triggered release or mild hyperthermia. Outcomes include apoptosis or reversion of activated stellate cells with downregulation of TGF beta SMAD3. *Abbreviations:* PMF, pulsed magnetic fields; HSC, hepatic stellate cell; SPION, superparamagnetic iron oxide nanoparticle; MAPK, mitogen-activated protein kinase; YAP, yes-associated protein; TAZ, transcriptional coactivator with PDZ binding motif; SMAD, mothers against decapentaplegic homolog; ETA, endothelin receptor type A (arrow down—decrease) (Created in BioRender. Kulbacka, J. (2025) https://BioRender.com/6povx4k).

Pulsed electromagnetic fields (PEMF) improved function in large-animal studies and decreased post-infarction scar burden in cardiology models, indicating a disease-modifying signal as opposed to the purely analgesic effects noted in musculoskeletal indications (Figure 2A). Modification of fibroblast phenotype, nitric oxide, and calcium signaling are among the hypothesized mechanisms. The preclinical signal is worth investigating as a supplement to guideline-directed therapy, even though human cardiac data are still pending [89]. In 2024–2025, research reviews confirm PEMF safety and bioactivity across various tissues (pain, bone, and neuromuscular), providing a translational safety scaffold for organ-specific fibrosis trials despite the lack of clinical validation for antifibrotic endpoints. For upcoming fibrosis research, parameterization (frequency, duty cycle, exposure time) is still variable [90]. Moreover, it has been shown that gradient or rotating magnetic fields can reduce TGF-β/SMAD3 signaling in solid tumor models and affect kidney injury-to-fibrosis transitions, involving mechanotransduction and MAPK pathways similar to those involved in fibrogenesis. These datasets demonstrate biologic plausibility that field exposure can direct profibrotic signaling networks, particularly TGF-β, even though they are not yet organ-fibrosis trials [91]. This technology opens new opportunities, but it also has some limitations. The available data indicate that device-based magnetic therapies have the potential to work in tandem with pharmaceuticals and provide repeatable, non-systemic dosing. However, before broad acceptance, rigorous dose-finding (flux density, gradients, temporal patterns) and standardized endpoints (e.g., MRI T1ρ, shear-wave elastography, quantitative collagen cross-linking) will be necessary [90].

### 5.2. Magnetic Nanocarriers for Targeted Antifibrotic Delivery

Recently, vitamin A retinoid chemistry has been used to target hepatic stellate cells (HSCs). When activated, HSCs store retinoids and upregulate specific receptors; retinoid-decorated nanoparticles exploit this property to deliver antifibrotic cargos. BMS-986263, a retinoid-conjugated lipid nanoparticle carrying siRNA against heat-shock protein-47 (HSP47—a collagen chaperone), demonstrated target engagement and histologic improvement in a Phase 2 study of patients with HCV-cured advanced fibrosis and favorable Phase 1 safety in hepatic impairment. The ineffectiveness of a subsequent Phase 2 program in MASH cirrhosis, however, underscores the difficulty of translating stromal gene silencing when considering disease stage and etiology [92]. In addition to HSP47 siRNA, retinoic acid-modified carriers (such as galangin-loaded particles) can also transport small molecules to HSCs, enhancing on-target exposure and antifibrotic efficacy in preclinical models. For combination cargoes (siRNA + TGF-β pathway inhibitors), the targeted platform—HSCs plus payload-agnostic vehicles—seems promising [93]. There are also interesting multipurpose SPIONs that serve as guided carriers. External magnets can guide SPIONs into fibrotic beds, where they can be functionalized with ligands to exhibit cell-type specificity (Figure 2B). In vivo research demonstrates that combining endothelin-A receptor antagonists with SPIONs enhances therapeutic efficacy. This approach can be easily applied to the fibrotic vasculature and interstitium, where endothelin signaling and extracellular matrix deposition meet. Additionally, these systems allow for the modulation of local cytokine gradients through mild hyperthermia or magnetically triggered drug release [94].

It was also observed that fibroblasts can be controlled magnetomechanically. Cytoskeletal fibers and focal adhesions can be mechanically disturbed by ultra-small iron oxide nanoparticles activated by low-frequency rotating fields. This results in stromal normalization and selective ablation of cancer-associated fibroblasts. If targeting is accurate, applying this principle to organ fibrosis may enable focal debulking of myofibroblasts while preserving parenchyma. To rewire fibroblast fate, new “magnetogenetic” tools also enable on-demand activation of mechano- and thermosensitive channels. Despite their early stages, these methods suggest a physical–biological lever that can be programmed to prevent fibrosis [95].

## 6. Future Directions and Challenges

### 6.1. Integration of Multi-Omics Approaches for Personalized Therapy

Researchers use a multi-omics approach to study fibrosis and identify new therapeutic targets. In the case of idiopathic pulmonary fibrosis, Multi-Omics Factor Analysis (MOFA) revealed key co-variation factors. Factor 1 distinguishes IPF from non-diseased tissues and is linked with disease stage, histological scores, and age. It highlights decreased surfactant signals, increased TGF-β, EMT-related genes, and markers of oxidative stress and mitochondrial dysfunction. Factor 2 differentiates early and late IPF stages, following a nonlinear pattern. It revealed increased ciliogenesis genes in later stages and identified mitochondrial dysfunction and lipid metabolism changes as critical components. These findings suggest potential biomarkers for early IPF detection and underscore the value of multi-omics in understanding fibrosis progression [96]. Furthermore, it was demonstrated that the integration of transcriptomic and proteomic data from blood enabled the identification of clinically significant molecular endotypes of IPF [97].

The study, using a multi-omics approach, revealed that the aggrephagy score (a selective form of autophagy) was significantly higher in the liver fibrosis group than in the normal group and was positively correlated with several metabolic pathways. An aggrephagy-related diagnostic model demonstrated greater efficiency than other liver fibrosis markers. Experiments showed that the removal of aggregates in liver fibrosis relied on aggrephagy. Single-cell data indicated that aggrephagy scores and cystic fibrosis transmembrane conductance regulator (CFTR) levels were predominantly located in hepatocytes. Additionally, the high-aggrephagy-score group exhibited increased cell–cell interaction strength, intercellular receptor-ligand signaling, and HNF1B transcription factor activity compared to the low-score group. Thus, targeting aggrephagy could be promising for treating liver fibrosis. Moreover, the results indicate that the aggrephagy score is a promising indicator in the diagnosis of liver fibrosis [98].

Regarding renal fibrosis, classifying kidney diseases by molecular mechanisms can enhance patient outcomes through targeted therapies. Omics data from kidney biopsies can identify disease mechanisms and prognostic biomarkers, potentially leading to non-invasive urinary markers reflecting kidney molecular pathways. Clinical trials have shown success in targeting molecular pathways by integrating various datasets. Combining targeted therapies with predictive biomarkers positions nephrology to implement precision medicine, ensuring the right treatment for the right patient at the right time [99].

### 6.2. Development of Combination Therapies Targeting Multiple Pathways

Developing combination therapies targeting multiple pathways in organ fibrosis and involving physical methods, such as magnetic fields or nanoparticles, is a promising approach given the complex, multifactorial nature of the disease. Organ fibrosis involves various cellular and molecular pathways, making single-target therapies often insufficient [100,101]. Combination therapies can offer a more effective treatment strategy by simultaneously addressing multiple aspects of fibrosis [102]. Complex therapies for liver fibrosis that target particularly cell interactions, soluble mediators, the ECM, and intracellular signaling hold promise for future treatments. However, these therapies need extensive preclinical testing, which will require significant resources. Effective combinations of drugs could inhibit fibrogenesis, promote fibrolysis, or target different cell types. Advancing combination therapies in clinical settings demands improved non-invasive biomarkers and technologies for measuring fibrosis and fibrogenesis. Personalized treatment plans for liver fibrosis or cirrhosis will also depend on these biomarkers, allowing for medication and dosage adjustments based on measurable treatment effects [103]. In patients with chronic alcoholic liver diseases, combining ursodeoxycholic acid (UDCA) with the angiotensin II receptor blocker candesartan improved fibrosis scores more than UDCA alone. However, since other clinical trials have not shown clear benefits, more research is needed to confirm the effectiveness of renin–angiotensin system antagonists in treating liver fibrosis [104].

In the case of Idiopathic Pulmonary Fibrosis, combined therapy with nintedanib and pirfenidone appears to be a promising research direction. However, there is some uncertainty regarding the combined use of these drugs due to their different mechanisms of action and unclear pharmacokinetic interactions [105]. Two clinical trials have explored this combination. Although these studies were not designed to fully assess efficacy, they hinted at the potential benefit of this combination [106,107]. Nevertheless, more comprehensive studies are needed to confirm these findings [105]. Multimodal treatments not only improve treatment results but also help reduce the side effects of the drugs used. Farnesoid X receptor (FXR) is a nuclear receptor highly expressed in the liver and intestines, playing a critical role in bile acid signaling and the regulation of inflammatory pathways. FXR activation reduces pro-inflammatory cytokines, inhibits inflammasome activation, and upregulates anti-inflammatory mediators. Studies indicate that FXR activation in hepatic stellate cells (HSCs) diminishes their response to profibrotic signals, such as TGF-β, thereby reducing extracellular matrix (ECM) formation and fibrosis development. FXR agonists, such as obeticholic acid (OCA) and cilofexor, show potential in treating fibrosis. However, OCA therapy is limited by dyslipidemia, specifically elevated low-density lipoprotein cholesterol (LDL-C), which increases the risk for non-alcoholic steatohepatitis patients with atherosclerosis [108]. Combining OCA with atorvastatin, an HMGR inhibitor, can mitigate this LDL-C elevation, as demonstrated in clinical research [109]. The most promising approaches, however, are logical combinations, such as PEMF to change fibroblast tone and SPION-guided siRNA or endothelin antagonist delivery; LOX inhibition to soften extracellular matrix (ECM) in conjunction with targeted TGF-β pathway blockade; and, in the heart, staged application of CAR-T with device-based surveillance. A specially controllable “dial” for timing and localization is provided by magnetic actuation [94].

Therapeutic strategies (Table 4) for organ fibrosis target key molecular and cellular pathways involved in fibrogenesis. These approaches aim to slow, halt, or reverse fibrosis across various organs by interfering with profibrotic signaling, modulating immune responses, and promoting tissue repair. The table below summarizes strategies that have been recently used.

Therapeutic strategies for organ fibrosis are very complex, targeting a range of molecular, cellular, and immune pathways. While some approaches, such as TGF-β inhibition and tyrosine kinase receptor blockade, have reached clinical use in specific fibrotic diseases, many others remain in preclinical or early clinical development. The multifactorial and organ-specific nature of fibrosis underscores the need for continued research into multimodal and personalized therapies, as well as a deeper understanding of the underlying mechanisms to improve patient outcomes [112,113,114,122,123].

### 6.3. Optimization of Drug Delivery Systems for Enhanced Efficacy and Specificity

Heterogeneity of patients and fibrosis in various organs, in addition to a lack of disease-specific biomarkers, are the main obstacles slowing the development of antifibrotic drugs [3]. However, two available medications were clinically approved for idiopathic pulmonary fibrosis—pirfenidone and nintedanib. Additionally, “both new and repurposed pharmacological treatments are now available for fibrosis, including those which inhibit each of the renin–angiotensin–aldosterone system (RAAS, e.g., ACEi, ARB, MRA and ARNi), galectin 3 and C-C motif chemokine receptors CCR2 and CCR5, as well as farnesoid X receptor and peroxisome proliferator-activated receptor (PPAR) agonists, modern antidiabetic agents and potentially other more recent antifibrotic interventions such as well as pirfenidone” [124].

To optimize drug delivery systems for enhanced efficacy and specificity, it is believed that the best approach is to view fibrosis as a pathological process distinct from inflammation. Because of the multi-stage progression of the disease, multimodal therapies should be tested. As a general therapeutic strategy for fibrosis, scientists suggest modulating macrophage activation status [3]. Due to the heterogeneity of macrophages, identifying the exact mechanism underlying their phenotypic transformation in fibrosis will enable the development of more specific drugs with fewer side effects [125]. Myofibroblasts are crucial cells in the final stage of fibrosis; thus, inhibiting their activity may have an antifibrotic effect. They are activated by various cytokines, including TGF-β1, which is directly linked to IL-17A, so therapeutic antibodies to TGF-β1 and antibodies that disrupt IL-17 signaling might also benefit treatment. Moreover, there are a few promising strategies for effective antifibrotic drugs, including targeting key epigenetic modifications or miRNAs, altering the metabolism of local tissue mesenchymal cells, targeting Treg cells or distinct macrophage populations, and targeting GPCRs expressed on the surfaces of multiple cell types [3,124]. Studies also implicate a special role of melatonin in the regulation of fibrosis. The pineal hormone regulates each stage of the disease and modulates several molecular pathways involved in inflammation, oxidative stress, and cellular injury. It also reduces fibrosis in the studied organs, but its effect will be more beneficial when combined with other antifibrotic agents [126].

All new approaches still suggest that a more integrated antifibrotic strategy is needed. Targeting key inflammatory mediators, profibrotic cytokines, and epigenetic and cell- and/or tissue-intrinsic changes may be the most effective way to treat this highly progressive and harmful disease. Future studies should focus on ways to slow or reverse fibrosis while simultaneously regenerating normal tissues, altering the immune response rather than destroying it, and modulating the fibrotic response to improve patients’ quality of life.

### 6.4. Addressing Issues Related to Patient Heterogeneity and Disease Progression

The difficulties in creating a successful universal antifibrotic drug are associated with patient heterogeneity and the usually slow progression of fibrosis [4]. High heterogeneity and functional diversity of macrophages and fibroblasts, both within and between organs, create obstacles in treating the disease. Firstly, macrophages polarize into distinct phenotypes, such as the pro-inflammatory M1 and the anti-inflammatory M2. Studies show that M2 macrophages promote myofibroblast formation to promote pulmonary fibrosis, especially M2a and M2c, by secreting TGF-β and pro-fibrotic factors. Moreover, the diversity of liver macrophages and myofibroblasts hinders the development of a new strategy against the disease. Additionally, heterogeneous fibroblasts can switch phenotypes during the progression and regression of fibrosis [4,125,127]. Studies also show that increasing age results in functional heterogeneity among fibroblasts, which must be considered when developing personalized drugs for elderly communities [4]. Furthermore, research indicates that telomere shortening contributes to the development of pulmonary fibrosis in adults and in familial forms. Other factors affecting the progression of organ fibrosis include environmental pollutants, chemicals, and microbes; inherited genetic disorders; persistent infections; chronic autoimmune inflammation; high serum cholesterol; obesity; and poorly controlled diabetes and hypertension [3,4]. It is worth noting the dysbiosis of human microbes that influences liver fibrosis. Transportation of bacteria and their products (ex., Lipopolysaccharide) through the intestinal barrier leads them to the portal vein and causes inflammation that induces fibrosis [4].

## 7. Conclusions

The continuous development and success of innovative therapies for organ fibrosis depend heavily on sustained research and collaboration. Thus, interdisciplinary efforts involving researchers, clinicians, and pharmaceutical companies are crucial for discovering new therapeutic targets and validating emerging treatments. Collaborative projects and shared databases enhance our understanding of fibrosis at the molecular level, leading to more accurate and effective therapies. Ongoing research initiatives and clinical trials are essential for translating laboratory findings into real-world clinical applications, ensuring that patients benefit from the latest scientific advancements. Antifibrotic medicines can now be more precisely controlled using magnetic nanocarriers and magnetic field-based therapies. While recent clinical programs (integrins, CTGF, and HSP47) have strengthened expectations, emphasized patient selection, and mechanistically defined endpoints, preclinical evidence for PEMF and magnetically actuated nanoparticles is still developing. The special spatiotemporal control that magnetism provides should be utilized in the upcoming generation of trials, ideally in adaptive designs that identify the optimal field parameters and delivery kinetics in tandem with clinical outcomes.

The ultimate goal of innovative fibrosis therapies is to improve outcomes and quality of life for patients with fibrotic disorders. By addressing the root causes of fibrosis and providing more effective treatments, patients can experience reduced symptoms, slower disease progression, and potentially even disease reversal. These advancements promise not only to extend life expectancy but also to enhance the overall quality of life for patients. The integration of precision medicine and advanced therapeutic approaches provides a pathway to more personalized care, offering patients tailored treatments that are more likely to be effective based on their unique genetic and molecular profiles.

## Figures and Tables

**Table 1:** Non-classical mechanisms and therapeutic strategies.

Mechanism	Key Features/Pathways	Therapeutic Strategies	Ref.
**Metabolic Reprogramming**	Glycolysis, lipid/amino acid metabolism, mitochondrial dysfunction, lactate lactylation	Glycolytic enzyme inhibitors, mitochondrial repair, metabolic pathway modulators	Preclinical/experimental [36,40,41,46]
**Epigenetic Regulation**	DNA methylation, histone modification, ncRNAs, m6A modification	DNMT/HDAC inhibitors, ncRNA/CRISPR-based editing, m6A targeting	Preclinical, limited clinical translation [38,43,48,50,53,54]

**Table 2:** Summary of innovative therapeutic approaches.

Drug/Method Name	Mechanism	Class	Target Disease	Phase	Reference
Nintedanib	Inhibitor of differentiation of fibroblasts into myofibroblasts, their migration and proliferation	Small molecule	Idiopathic pulmonary fibrosis (IPF)	Approved	[59,60]
Pirfenidone	Inhibitor of TGF-β	Small molecule	Idiopathic pulmonary fibrosis (IPF)	Approved	[59,60]
Lisinopril	Inhibitor of angiotensin converting enzyme -> inhibitor of angiotensin II and TGF-β1	Small molecule	Idiopathic pulmonary fibrosis (IPF), an organ fibrosis	Research	[60]
Metformin	Activation of AMPK pathways, inhibition of TGF-β signaling, reduction in collagen and fibronectin production, deactivation of myofibroblasts, and suppression of macrophage and fibroblast activation	Small molecule	Idiopathic pulmonary fibrosis (IPF), an organ fibrosis	Research	[60]
INS018_055	TNIK inhibitor	Small molecule inhibitor generated by AI-based design	Organ fibrosis	Completed Phase I clinical trials	[75]
Pamrevlumab	Human antibody against CTGF	Monoclonal antibody	Idiopathic pulmonary fibrosis (IPF)	Phase 3 RCT	[63,64]
BG00011	Anti-αvβ6 integrin monoclonal antibody	Monoclonal antibody	Idiopathic pulmonary fibrosis (IPF)	2a randomized, placebo-controlled trial	[63,64]
Lebrikizumab	Monoclonal antibody to IL-13	Monoclonal antibody	Idiopathic pulmonary fibrosis (IPF)	Research	[3,4]
Therapeutic antibodies to TGF-β1	activates myofibroblasts and humanized monoclonal antibody targeting lysyl oxidase-like-2 (catalyzes the cross-linking of collagen)	Monoclonal antibody	cardiac fibrosis, IPF and liver fibrosis	Clinical trial	[65]
Human monoclonal antibody to CCL2	Recruits inflammatory monocytes	Monoclonal antibody	Organ fibrosis	Phase 1	[65]
AAV9-Tspyl2 gene therapy	Restores cell division autoantigen-1 (CDA1) expression	Gene therapy	Renal fibrosis	Research	[66]
CRISPR/dCas9 system	Acts on liver fibrosis effector cells	Gene therapy	Liver fibrosis	Research	[67]
Pluripotent stem cells (iPSCs)	Differentiates into specific cell types, blocks the TGF-β/Smad pathway	Cell	Organ fibrosis	Research	[69,70,71,72,73,74]

*Abbreviations:* AAV9-Tspyl2, Adeno-Associated Virus 9 carrying Testis-Specific Protein Y-like 2; AMPK, Adenosine Monophosphate-Activated Protein Kinase; CCL2, Chemokine (C-C Motif) Ligand 2; CDA1, Cell Division Autoantigen 1; CRISPR/dCas9, Clustered Regularly Interspaced Short Palindromic Repeats; CTGF, Connective Tissue Growth Factor; IL-13, Interleukin-13; IPF, Idiopathic Pulmonary Fibrosis; RCT, Randomized Controlled Trial; TGF-β, Transforming Growth Factor Beta; TNIK, TRAF2- and NCK-Interacting Kinase.

**Table 3:** Summary of preclinical and clinical studies.

Substance/Treatment	Key Findings and Effects	References
Pirfenidone	Reduces inflammation, fibroblast activity, and collagen deposition; improves alveolar structure	[76,79]
Gene-Modified MSCs	Enhance anti-inflammatory effects, reduce fibrosis markers, and promote tissue regeneration	[77]
AD-MSCs and Conditioned Medium	Reduce inflammation and fibrosis markers; CM is slightly more effective than MSCs	[78]
Anti-TGF-β Antibodies	Fresolimumab and similar antibodies show no significant improvement in fibrosis outcomes	[80,81]
Microneedling + CM	Improves skin thickness and density; increases patient satisfaction	[82]
BM-MSCs and UC-MSCs (Liver Use)	Improve liver function in fibrosis and cirrhosis; minimal tumorigenesis risk	[83,84,85,86]

*Abbreviations:* AD-MSCs, Adipose-Derived Mesenchymal Stem Cells; BM-MSCs, Bone Marrow Mesenchymal Stem Cells; CM, Conditioned Medium; FVC, Forced Vital Capacity; MSCs, Mesenchymal Stem Cells; TNF-α, Tumor Necrosis Factor Alpha; TGF-β, Transforming Growth Factor Beta; UC-MSCs, Umbilical Cord Mesenchymal Stem Cells.

**Table 4:** Therapeutic strategies as future directions for organ fibrosis.

Strategy/Target	Mechanism/Pathway	Organ/System(s)	Status/Notes
**TGF-β inhibitors**	TGF-β signaling	Multiple	Developed, some approved [32,54,110,111,112]
**RAAS blockers, antioxidants**	Profibrotic/inflammatory	Kidney, others	Under investigation [113,114]
**NLRP3 inflammasome inhibitors**	Inflammation	Ovary	Developing [112]
**Ferroptosis pathway inhibitors**	Lipid peroxidation/cell death	Lung, heart, liver, kidney	Promising, drugs identified [115,116,117]
**Piezo channel inhibitors**	Mechanosensitive signaling	Multiple	Basic research stage [118]
**Wnt pathway inhibitors**	Wnt/β-catenin signaling	Liver	Preclinical/clinical [45]
**IL-13/IL-4 pathway inhibitors**	Immune modulation	Lung, IBD, others	Mixed results [119]
**ECM degradation, myofibroblast elimination**	ECM remodeling, cell targeting	Multiple	Clinical trials ongoing [112,114]
**TXNDC5 deletion**	Molecular target	Multiple	Potential strategy [120]
**p53 targeting**	Cell-type specific modulation	Multiple	Investigational [121]
**miRNA-based therapies**	Gene regulation	Multiple	Emerging [113,117]

## Abbreviations

The following abbreviations are used in this manuscript:

ACEi	Angiotensin-Converting Enzyme Inhibitor
AD-MSCs	Adipose-derived Mesenchymal Stem Cells
ARB	Angiotensin II Receptor Blocker
BM-MSCs	Bone Marrow Mesenchymal Stem Cells
BLM-induced PF	Bleomycin-induced Pulmonary Fibrosis
CCL	C-C Motif Chemokine Ligand
CLDN1	Claudin-1
CRISPR/dCas9	Clustered Regularly Interspaced Short Palindromic Repeats/Dead Cas9
CTGF	Connective Tissue Growth Factor
ECM	Extracellular Matrix
EMT	Epithelial-to-Mesenchymal Transition
EpCAM	Epithelial Cell Adhesion Molecule
ESDL	End-Stage Liver Disease
FVC	Forced Vital Capacity
FXR	Farnesoid X Receptor
HBV	Hepatitis B Virus
HCV	Hepatitis C Virus
HLFs	Human Lung Fibroblasts
HPS1	Hermansky–Pudlak Syndrome 1
ILD	Interstitial Lung Disease
IPF	Idiopathic Pulmonary Fibrosis
iPSCs	Induced Pluripotent Stem Cells
LDL-C	Low-Density Lipoprotein Cholesterol
MMPs	Matrix Metalloproteinases
MOFA	Multi-Omics Factor Analysis
MSC	Mesenchymal Stem Cell
OCA	Obeticholic Acid
PCNA	Proliferating Cell Nuclear Antigen
PF	Pulmonary Fibrosis
PFD	Pirfenidone
PPAR	Peroxisome Proliferator-Activated Receptor
RAAS	Renin–Angiotensin–Aldosterone System
R-Smads	Receptor-regulated Smad proteins
TGF-β	Transforming Growth Factor Beta
TGF-βR	Transforming Growth Factor Beta Receptor
Th2	T-helper 2
TIMPs	Tissue Inhibitors of Metalloproteinases
TNF-α	Tumor Necrosis Factor Alpha
TNIK	Traf2- and Nck-interacting kinase
UC-MSCs	Umbilical Cord-derived Mesenchymal Stem Cells
UDCA	Ursodeoxycholic Acid
VCAM-1	Vascular Cell Adhesion Molecule-1

## References

[1] Lurje I., Gaisa N.T., Weiskirchen R., Tacke F. (2023). Mechanisms of Organ Fibrosis: Emerging Concepts and Implications for Novel Treatment Strategies. Mol. Asp. Med..

[2] Parola M., Pinzani M. (2019). Pathophysiology of Organ and Tissue Fibrosis. Mol. Asp. Med..

[3] Wynn T.A., Ramalingam T.R. (2012). Mechanisms of Fibrosis: Therapeutic Translation for Fibrotic Disease. Nat. Med..

[4] Henderson N.C., Rieder F., Wynn T.A. (2020). Fibrosis: From Mechanisms to Medicines. Nature.

[5] Shivpuri A., Sharma S., Trehan M., Hivpuri A.S. (2013). Oral Submucous Fibrosis in a 10 Year Old Girl—Case Report. Dent. Med. Probl..

[6] DeLeon-Pennell K.Y., Barker T.H., Lindsey M.L. (2020). Fibroblasts: The Arbiters of Extracellular Matrix Remodeling. Matrix Biol..

[7] Lee K.A., Nelson C.M. (2012). New Insights into the Regulation of Epithelial–Mesenchymal Transition and Tissue Fibrosis. Int. Rev. Cell Mol. Biol..

[8] Rockey D.C., Bell P.D., Hill J.A. (2015). Fibrosis—A Common Pathway to Organ Injury and Failure. N. Engl. J. Med..

[9] Dees C., Chakraborty D., Distler J.H.W. (2021). Cellular and Molecular Mechanisms in Fibrosis. Exp. Dermatol..

[10] Braga T.T., Agudelo J.S.H., Camara N.O.S. (2015). Macrophages During the Fibrotic Process: M2 as Friend and Foe. Front. Immunol..

[11] Kendall R.T., Feghali-Bostwick C.A. (2014). Fibroblasts in Fibrosis: Novel Roles and Mediators. Front. Pharmacol..

[12] Griffin M.F., desJardins-Park H.E., Mascharak S., Borrelli M.R., Longaker M.T. (2020). Understanding the Impact of Fibroblast Heterogeneity on Skin Fibrosis. Dis. Model. Mech..

[13] Damerau A., Rosenow E., Alkhoury D., Buttgereit F., Gaber T. (2024). Fibrotic Pathways and Fibroblast-like Synoviocyte Phenotypes in Osteoarthritis. Front. Immunol..

[14] Bayreuther K., Rodemann H.P., Francz P.I., Maier K. (1988). Differentiation of Fibroblast Stem Cells. J. Cell Sci. Suppl..

[15] Antar S.A., Ashour N.A., Marawan M.E., Al-Karmalawy A.A. (2023). Fibrosis: Types, Effects, Markers, Mechanisms for Disease Progression, and Its Relation with Oxidative Stress, Immunity, and Inflammation. Int. J. Mol. Sci..

[16] Flannigan K.L., Nieves K.M., Szczepanski H.E., Serra A., Lee J.W., Alston L.A., Ramay H., Mani S., Hirota S.A. (2023). The Pregnane X Receptor and Indole-3-Propionic Acid Shape the Intestinal Mesenchyme to Restrain Inflammation and Fibrosis. Cell. Mol. Gastroenterol. Hepatol..

[17] Laudadio I., Carissimi C., Scafa N., Bastianelli A., Fulci V., Renzini A., Russo G., Oliva S., Vitali R., Palone F. (2024). Characterization of Patient-Derived Intestinal Organoids for Modelling Fibrosis in Inflammatory Bowel Disease. Inflamm. Res..

[18] Ma X., Li J., Li M., Qi G., Wei L., Zhang D. (2024). Nets in Fibrosis: Bridging Innate Immunity and Tissue Remodeling. Int. Immunopharmacol..

[19] Horsburgh S., Todryk S., Ramming A., Distler J.H.W., O’Reilly S. (2018). Innate Lymphoid Cells and Fibrotic Regulation. Immunol. Lett..

[20] Jiang Y., Cai R., Huang Y., Zhu L., Xiao L., Wang C., Wang L. (2024). Macrophages in Organ Fibrosis: From Pathogenesis to Therapeutic Targets. Cell Death Discov..

[21] Iwanaga T., Chiba T., Nakamura M., Kaneko T., Ao J., Qiang N., Ma Y., Zhang J., Kogure T., Yumita S. (2023). Miglustat, a Glucosylceramide Synthase Inhibitor, Mitigates Liver Fibrosis through TGF-β/Smad Pathway Suppression in Hepatic Stellate Cells. Biochem. Biophys. Res. Commun..

[22] Wang L., Wang S., Lin J., Li J., Wang M., Yu J., Sun J., Tang N., Jiao C., Ma J. (2025). Treg and Intestinal Myofibroblasts-Derived Amphiregulin Induced by TGF-β Mediates Intestinal Fibrosis in Crohn’s Disease. J. Transl. Med..

[23] Jasso G.J., Jaiswal A., Varma M., Laszewski T., Grauel A., Omar A., Silva N., Dranoff G., Porter J.A., Mansfield K. (2022). Colon Stroma Mediates an Inflammation-Driven Fibroblastic Response Controlling Matrix Remodeling and Healing. PLoS Biol..

[24] Martín-Taboada M., Corrales P., Medina-Gómez G., Vila-Bedmar R. (2022). Tackling the Effects of Extracellular Vesicles in Fibrosis. Eur. J. Cell Biol..

[25] Ma C., Li M., Hou P., Wang X., Sun Z., Li Y., Xiao Y., Li Y. (2024). Biofilm Dynamic Changes in Drip Irrigation Emitter Flow Channels Using Reclaimed Water. Agric. Water Manag..

[26] Duan S., Kanda H., Zhu F., Okubo M., Koike T., Ohno Y., Tanaka T., Harima Y., Miyamichi K., Fukui H. (2025). Sympathetic Overactivation Drives Colonic Eosinophil Infiltration Linked to Visceral Hypersensitivity in Irritable Bowel Syndrome. Cell. Mol. Gastroenterol. Hepatol..

[27] Enzerink A., Salmenperä P., Kankuri E., Vaheri A. (2009). Clustering of Fibroblasts Induces Proinflammatory Chemokine Secretion Promoting Leukocyte Migration. Mol. Immunol..

[28] Li Y., Wu W., Liu Q., Wu Q., Ren P., Xi X., Liu H., Zhao J., Zhang W., Wang Z. (2024). Specific Surface-Modified Iron Oxide Nanoparticles Trigger Complement-Dependent Innate and Adaptive Antileukaemia Immunity. Nat. Commun..

[29] Hsu T., Nguyen-Tran H.-H., Trojanowska M. (2019). Active Roles of Dysfunctional Vascular Endothelium in Fibrosis and Cancer. J. Biomed. Sci..

[30] Choi S., Jung H.J., Kim M.W., Kang J.-H., Shin D., Jang Y.-S., Yoon Y.S., Oh S.H. (2019). A Novel STAT3 Inhibitor, STX-0119, Attenuates Liver Fibrosis by Inactivating Hepatic Stellate Cells in Mice. Biochem. Biophys. Res. Commun..

[31] Meng X.M., Nikolic-Paterson D.J., Lan H.Y. (2016). TGF-β: The Master Regulator of Fibrosis. Nat. Rev. Nephrol..

[32] Muñoz-Félix J.M., González-Núñez M., López-Novoa J.M. (2013). ALK1-Smad1/5 Signaling Pathway in Fibrosis Development: Friend or Foe?. Cytokine Growth Factor Rev..

[33] Lee J., An J.N., Hwang J.H., Lee H., Lee J.P., Kim S.G. (2019). P38 MAPK Activity Is Associated with the Histological Degree of Interstitial Fibrosis in IgA Nephropathy Patients. PLoS ONE.

[34] Wei Q., Gan C., Sun M., Xie Y., Liu H., Xue T., Deng C., Mo C., Ye T. (2024). BRD4: An Effective Target for Organ Fibrosis. Biomark. Res..

[35] Li M., Chen B., Zhang X., Zhuo T., Liu X. (2025). Advances in Novel Therapeutics for Idiopathic Pulmonary Fibrosis. Pulm. Pharmacol. Ther..

[36] Wang W., Dai R., Cheng M., Chen Y., Gao Y., Hong X., Zhang W., Wang Y., Zhang L. (2024). Metabolic Reprogramming and Renal Fibrosis: What Role Might Chinese Medicine Play?. Chin. Med..

[37] Page A., Mann D.A. (2015). Epigenetic Regulation of Liver Fibrosis. Clin. Res. Hepatol. Gastroenterol..

[38] Ulukan B., Sila Ozkaya Y., Zeybel M. (2019). Advances in the Epigenetics of Fibroblast Biology and Fibrotic Diseases. Curr. Opin. Pharmacol..

[39] Feng L., Chen X., Huang Y., Zhang X., Zheng S., Xie N. (2023). Immunometabolism Changes in Fibrosis: From Mechanisms to Therapeutic Strategies. Front. Pharmacol..

[40] Zhu X., Jiang L., Long M., Wei X., Hou Y., Du Y. (2021). Metabolic Reprogramming and Renal Fibrosis. Front. Med..

[41] Renaud L., da Silveira W.A., Takamura N., Hardiman G., Feghali-Bostwick C. (2020). Prominence of IL6, IGF, TLR, and Bioenergetics Pathway Perturbation in Lung Tissues of Scleroderma Patients With Pulmonary Fibrosis. Front. Immunol..

[42] Ung C.Y., Onoufriadis A., Parsons M., McGrath J.A., Shaw T.J. (2021). Metabolic Perturbations in Fibrosis Disease. Int. J. Biochem. Cell Biol..

[43] Li J., Wang S., Yuan J., Mao X., Wang X., Zhang L., Dong Q., Chen Z., Wang Y., Tang N. (2025). Tissue Regeneration: Unraveling Strategies for Resolving Pathological Fibrosis. Cell Stem Cell.

[44] Li X., Zhang Q., Wang Z., Zhuang Q., Zhao M. (2021). Immune and Metabolic Alterations in Liver Fibrosis: A Disruption of Oxygen Homeostasis?. Front. Mol. Biosci..

[45] Ji P., Li Y., Wang Z., Jia S., Jiang X., Chen H., Wang Q. (2024). Advances in Precision Gene Editing for Liver Fibrosis: From Technology to Therapeutic Applications. Biomed. Pharmacother..

[46] Lu P., Liu M., Zhang L., Fan J.-J., Fu Q., Zhang J., Sun Y. (2026). Molecular Mechanisms and Targeted Intervention Strategies of Renal Tubular Epithelial Cell Glycolytic Reprogramming in Renal Fibrosis. Life Sci..

[47] Ligresti G., Raslan A.A., Hong J., Caporarello N., Confalonieri M., Huang S.K. (2023). Mesenchymal Cells in the Lung: Evolving Concepts and Their Role in Fibrosis. Gene.

[48] Dong Q.-Q., Yang Y., Tao H., Lu C., Yang J.-J. (2024). M6A Epitranscriptomic and Epigenetic Crosstalk in Liver Fibrosis: Special Emphasis on DNA Methylation and Non-Coding RNAs. Cell. Signal..

[49] McDonnell F., Irnaten M., Clark A.F., O’Brien C.J., Wallace D.M. (2016). Hypoxia-Induced Changes in DNA Methylation Alter RASAL1 and TGFβ1 Expression in Human Trabecular Meshwork Cells. PLoS ONE.

[50] You J.-B., Cao Y., You Q.-Y., Liu Z.-Y., Wang X.-C., Ling H., Sha J.-M., Tao H. (2024). The Landscape of Histone Modification in Organ Fibrosis. Eur. J. Pharmacol..

[51] Liu Y., Wen D., Ho C., Yu L., Zheng D., O’Reilly S., Gao Y., Li Q., Zhang Y. (2023). Epigenetics as a Versatile Regulator of Fibrosis. J. Transl. Med..

[52] Laduron S., Deplus R., Zhou S., Kholmanskikh O., Godelaine D., De Smet C., Hayward S.D., Fuks F., Boon T., De Plaen E. (2004). MAGE-A1 Interacts with Adaptor SKIP and the Deacetylase HDAC1 to Repress Transcription. Nucleic Acids Res..

[53] Singh P., Schimenti J.C., Bolcun-Filas E. (2015). A Mouse Geneticist’s Practical Guide to CRISPR Applications. Genetics.

[54] Chen S., Gao J., Chen J., Huang Y., Wang L., Lin J., Zhao M., Shen Y., Zhao G., Cheng Y. (2025). Roles and Potential Mechanisms of Hepatic Stellate Cells Activation Mediated by Epigenetics through Insulin like Growth Factor 1 and Receptor: A Review. Int. J. Biol. Macromol..

[55] Qiu Y., Ye W. (2025). Therapeutic Efficacy of Pirfenidone and Nintedanib in Pulmonary Fibrosis; a Systematic Review and Meta-Analysis. Ann. Thorac. Med..

[56] Meersseman C., Martínez Besteiro E., Romain-Scelle N., Crestani B., Marchand-Adam S., Nunes H., Wémeau-Stervinou L., Borie R., Diesler R., Valenzuela C. (2025). Nintedanib Combined With Pirfenidone in Patients With Idiopathic Pulmonary Fibrosis or Progressive Pulmonary Fibrosis: A Long-Term Retrospective Multicentre Study (Combi-PF). Arch. Bronconeumol..

[57] Yang M., Tan Y., Yang T., Xu D., Chen M., Chen L. (2025). Efficacy and Safety of Antifibrotic Drugs for Interstitial Lung Diseases Other than IPF: A Systematic Review, Meta-Analysis and Trial Sequential Analysis. PLoS ONE.

[58] Bargagli E., Piccioli C., Rosi E., Torricelli E., Turi L., Piccioli E., Pistolesi M., Ferrari K., Voltolini L. (2019). Pirfenidone and Nintedanib in Idiopathic Pulmonary Fibrosis: Real-Life Experience in an Italian Referral Centre. Pulmonology.

[59] Kim K.-I., Hossain R., Li X., Lee H.J., Lee C.J. (2023). Searching for Novel Candidate Small Molecules for Ameliorating Idiopathic Pulmonary Fibrosis: A Narrative Review. Biomol. Ther..

[60] Ma J., Li G., Wang H., Mo C. (2024). Comprehensive Review of Potential Drugs with Anti-Pulmonary Fibrosis Properties. Biomed. Pharmacother..

[61] Vistnes M. (2024). Hitting the Target! Challenges and Opportunities for TGF-β Inhibition for the Treatment of Cardiac Fibrosis. Pharmaceuticals.

[62] Tran T.N., Le P.H., Yang S., Luong T., Kim J. (2025). Rewiring the Scar: Translational Advances in Cardiac Fibrosis. Korean J. Physiol. Pharmacol..

[63] Sgalla G., Flore M., Siciliano M., Richeldi L. (2020). Antibody-Based Therapies for Idiopathic Pulmonary Fibrosis. Expert. Opin. Biol. Ther..

[64] Saito S., Alkhatib A., Kolls J.K., Kondoh Y., Lasky J.A. (2019). Pharmacotherapy and Adjunctive Treatment for Idiopathic Pulmonary Fibrosis (IPF). J. Thorac. Dis..

[65] Roehlen N., Saviano A., El Saghire H., Crouchet E., Nehme Z., Del Zompo F., Jühling F., Oudot M.A., Durand S.C., Duong F.H.T. (2022). A Monoclonal Antibody Targeting Nonjunctional Claudin-1 Inhibits Fibrosis in Patient-Derived Models by Modulating Cell Plasticity. Sci. Transl. Med..

[66] Zhang S., Tong X., Liu S., Huang J., Zhang L., Zhang T., Wang D., Fan H. (2023). AAV9-Tspyl2 Gene Therapy Retards Bleomycin-Induced Pulmonary Fibrosis by Modulating Downstream TGF-β Signaling in Mice. Cell Death Dis..

[67] Luo N., Zhong W., Li J., Lu J., Dong R. (2022). CRISPR/DCas9 for Hepatic Fibrosis Therapy: Implications and Challenges. Mol. Biol. Rep..

[68] Nieto-Alamilla G., Behan M., Hossain M., Gochuico B.R., Malicdan M.C.V. (2022). Hermansky-Pudlak Syndrome: Gene Therapy for Pulmonary Fibrosis. Mol. Genet. Metab..

[69] Cheng W., Fan C., Song Q., Chen P., Peng H., Lin L., Liu C., Wang B., Zhou Z. (2023). Induced Pluripotent Stem Cell-Based Therapies for Organ Fibrosis. Front. Bioeng. Biotechnol..

[70] Pouyanfard S., Meshgin N., Cruz L.S., Diggle K., Hashemi H., Pham T.V., Fierro M., Tamayo P., Fanjul A., Kisseleva T. (2021). Human Induced Pluripotent Stem Cell-Derived Macrophages Ameliorate Liver Fibrosis. Stem Cells.

[71] Yan Q., Quan Y., Sun H., Peng X., Zou Z., Alcorn J.L., Wetsel R.A., Wang D. (2014). A Site-Specific Genetic Modification for Induction of Pluripotency and Subsequent Isolation of Derived Lung Alveolar Epithelial Type II Cells. Stem Cells.

[72] Alvarez-Palomo B., Sanchez-Lopez L.I., Moodley Y., Edel M.J., Edel M.J., Edel M.J., Serrano-Mollar A., Serrano-Mollar A. (2020). Induced Pluripotent Stem Cell-Derived Lung Alveolar Epithelial Type II Cells Reduce Damage in Bleomycin-Induced Lung Fibrosis. Stem Cell Res. Ther..

[73] Povero D., Pinatel E.M., Leszczynska A., Goyal N.P., Nishio T., Kim J., Kneiber D., de Araujo Horcel L., Eguchi A., Ordonez P.M. (2019). Human Induced Pluripotent Stem Cell-Derived Extracellular Vesicles Reduce Hepatic Stellate Cell Activation and Liver Fibrosis. JCI Insight.

[74] Zhou Y., Zhang Q., Gao Y., Tan M., Zheng R., Zhao L., Zhang X. (2018). Induced Pluripotent Stem Cell-Conditioned Medium Suppresses Pulmonary Fibroblast-to-Myofibroblast Differentiation via the Inhibition of TGF-Β1/Smad Pathway. Int. J. Mol. Med..

[75] Ren F., Aliper A., Chen J., Zhao H., Rao S., Kuppe C., Ozerov I.V., Zhang M., Witte K., Kruse C. (2024). A Small-Molecule TNIK Inhibitor Targets Fibrosis in Preclinical and Clinical Models. Nat. Biotechnol..

[76] Lv Q., Wang J., Xu C., Huang X., Ruan Z., Dai Y. (2020). Pirfenidone Alleviates Pulmonary Fibrosis in Vitro and in Vivo through Regulating Wnt/GSK-3β/β-Catenin and TGF-Β1/Smad2/3 Signaling Pathways. Mol. Med..

[77] Yang X., Li Q., Liu W., Zong C., Wei L., Shi Y., Han Z. (2023). Mesenchymal Stromal Cells in Hepatic Fibrosis/Cirrhosis: From Pathogenesis to Treatment. Cell. Mol. Immunol..

[78] Felix R.G., Bovolato A.L.C., Cotrim O.S., Leão P.D.S., Batah S.S., Golim M.d.A., Velosa A.P., Teodoro W., Martins V., Cruz F.F. (2020). Adipose-Derived Stem Cells and Adipose-Derived Stem Cell-Conditioned Medium Modulate in Situ Imbalance between Collagen I- and Collagen V-Mediated IL-17 Immune Response Recovering Bleomycin Pulmonary Fibrosis. Histol. Histopathol..

[79] Behr J., Prasse A., Kreuter M., Johow J., Rabe K.F., Bonella F., Bonnet R., Grohe C., Held M., Wilkens H. (2021). Pirfenidone in Patients with Progressive Fibrotic Interstitial Lung Diseases Other than Idiopathic Pulmonary Fibrosis (RELIEF): A Double-Blind, Randomised, Placebo-Controlled, Phase 2b Trial. Lancet Respir. Med..

[80] Voelker J., Berg P.H., Sheetz M., Duffin K., Shen T., Moser B., Greene T., Blumenthal S.S., Rychlik I., Yagil Y. (2017). Anti-TGF-β 1 Antibody Therapy in Patients with Diabetic Nephropathy. J. Am. Soc. Nephrol..

[81] Vincenti F., Fervenza F.C., Campbell K.N., Diaz M., Gesualdo L., Nelson P., Praga M., Radhakrishnan J., Sellin L., Singh A. (2017). A Phase 2, Double-Blind, Placebo-Controlled, Randomized Study of Fresolimumab in Patients With Steroid-Resistant Primary Focal Segmental Glomerulosclerosis. Kidney Int. Rep..

[82] Behrangi E., Feizollahi M., Zare S., Goodarzi A., Ghasemi M.R., Sadeghzadeh-Bazargan A., Dehghani A., Nouri M., Zeinali R., Roohaninasab M. (2024). Evaluation of the Efficacy of Mesenchymal Stem Cells Derived Conditioned Medium in the Treatment of Striae Distensae: A Double Blind Randomized Clinical Trial. Stem Cell Res. Ther..

[83] Zhang S., Yang Y., Fan L., Zhang F., Li L. (2020). The Clinical Application of Mesenchymal Stem Cells in Liver Disease: The Current Situation and Potential Future. Ann. Transl. Med..

[84] Suk K.T., Yoon J.H., Kim M.Y., Kim C.W., Kim J.K., Park H., Hwang S.G., Kim D.J., Lee B.S., Lee S.H. (2016). Transplantation with Autologous Bone Marrow-Derived Mesenchymal Stem Cells for Alcoholic Cirrhosis: Phase 2 Trial. Hepatology.

[85] Salama H., Zekri A.R.N., Medhat E., Al Alim S.A., Ahmed O.S., Bahnassy A.A., Lotfy M.M., Ahmed R., Musa S. (2014). Peripheral Vein Infusion of Autologous Mesenchymal Stem Cells in Egyptian HCV-Positive Patients with End-Stage Liver Disease. Stem Cell Res. Ther..

[86] Zhang Z., Lin H., Shi M., Xu R., Fu J., Lv J., Chen L., Lv S., Li Y., Yu S. (2012). Human Umbilical Cord Mesenchymal Stem Cells Improve Liver Function and Ascites in Decompensated Liver Cirrhosis Patients. J. Gastroenterol. Hepatol..

[87] Staab-Weijnitz C.A. (2022). Fighting the Fiber: Targeting Collagen in Lung Fibrosis. Am. J. Respir. Cell Mol. Biol..

[88] Eftekhari A., Arjmand A., Asheghvatan A., Švajdlenková H., Šauša O., Abiyev H., Ahmadian E., Smutok O., Khalilov R., Kavetskyy T. (2021). The Potential Application of Magnetic Nanoparticles for Liver Fibrosis Theranostics. Front. Chem..

[89] Wang S., Pei G., Shen J., Fang Z., Chen T., Wang L., Cheng H., Li H., Pei H., Feng Q. (2025). Pulsed Electromagnetic Fields Treatment Ameliorates Cardiac Function after Myocardial Infarction in Mice and Pigs. J. Adv. Res..

[90] Koura G.M.R., Elshiwi A.M.F., Alshahrani M.S., Elimy D.A., Alshahrani R.A.N., Alfaya F.F., Alshehri S.H.S., Hadi A.A., Alshehri M.A., Alnakhli H.H. (2025). Effectiveness of Electromagnetic Field Therapy in Mechanical Low Back Pain: A Randomized Controlled Trial. J. Pain. Res..

[91] Zhang G., Yu T., Chai X., Zhang S., Liu J., Zhou Y., Yin D., Zhang C. (2024). Gradient Rotating Magnetic Fields Impairing F-Actin-Related Gene CCDC150 to Inhibit Triple-Negative Breast Cancer Metastasis by Inactivating TGF-Β1/SMAD3 Signaling Pathway. Research.

[92] Lawitz E.J., Shevell D.E., Tirucherai G.S., Du S., Chen W., Kavita U., Coste A., Poordad F., Karsdal M., Nielsen M. (2021). BMS-986263 in Patients with Advanced Hepatic Fibrosis: 36-week Results from a Randomized, Placebo-controlled Phase 2 Trial. Hepatology.

[93] Xiong Y., Wu B., Guo X., Shi D., Xia H., Xu H., Liu X. (2023). Galangin Delivered by Retinoic Acid-Modified Nanoparticles Targeted Hepatic Stellate Cells for the Treatment of Hepatic Fibrosis. RSC Adv..

[94] ten Hove M., Smyris A., Booijink R., Wachsmuth L., Hansen U., Alic L., Faber C., Höltke C., Bansal R. (2024). Engineered SPIONs Functionalized with Endothelin a Receptor Antagonist Ameliorate Liver Fibrosis by Inhibiting Hepatic Stellate Cell Activation. Bioact. Mater..

[95] Lopez S., Hallali N., Lalatonne Y., Hillion A., Antunes J.C., Serhan N., Clerc P., Fourmy D., Motte L., Carrey J. (2021). Magneto-Mechanical Destruction of Cancer-Associated Fibroblasts Using Ultra-Small Iron Oxide Nanoparticles and Low Frequency Rotating Magnetic Fields. Nanoscale Adv..

[96] Pattaroni C., Begka C., Cardwell B., Jaffar J., Macowan M., Harris N.L., Westall G.P., Marsland B.J. (2024). Multi-Omics Integration Reveals a Nonlinear Signature That Precedes Progression of Lung Fibrosis. Clin. Transl. Immunol..

[97] Ruan P., Todd J.L., Zhao H., Liu Y., Vinisko R., Soellner J.F., Schmid R., Kaner R.J., Luckhardt T.R., Neely M.L. (2023). Integrative Multi-Omics Analysis Reveals Novel Idiopathic Pulmonary Fibrosis Endotypes Associated with Disease Progression. Respir. Res..

[98] Chen J., Zhou Z.C., Yan Y., Wu S.Z., Ma T., Xuan H., Wang R.C., Gu C.Y., Liu Y.H., Liu Q.Q. (2024). Characterization of Aggrephagy-Related Genes to Predict the Progression of Liver Fibrosis from Multi-Omics Profiles. Biomed. Technol..

[99] Eddy S., Mariani L.H., Kretzler M. (2020). Integrated Multi-Omics Approaches to Improve Classification of Chronic Kidney Disease. Nat. Rev. Nephrol..

[100] Zhao M., Wang L., Wang M., Zhou S., Lu Y., Cui H., Racanelli A.C., Zhang L., Ye T., Ding B. (2022). Targeting Fibrosis, Mechanisms and Cilinical Trials. Signal Transduct. Target. Ther..

[101] Wuyts W.A., Antoniou K.M., Borensztajn K., Costabel U., Cottin V., Crestani B., Grutters J.C., Maher T.M., Poletti V., Richeldi L. (2014). Combination Therapy: The Future of Management for Idiopathic Pulmonary Fibrosis?. Lancet Respir. Med..

[102] Xu Y., Zhou X., Wang X., Jin Y., Zhou L., Ye J. (2024). Progress of Mesenchymal Stem Cells (MSCs) & MSC-Exosomes Combined with Drugs Intervention in Liver Fibrosis. Biomed. Pharmacother..

[103] Schuppan D., Kim Y.O. (2013). Evolving Therapies for Liver Fibrosis. J. Clin. Investig..

[104] Wang F.-D., Zhou J., Chen E.Q. (2022). Molecular Mechanisms and Potential New Therapeutic Drugs for Liver Fibrosis. Front. Pharmacol..

[105] Bonella F., Spagnolo P., Ryerson C. (2023). Current and Future Treatment Landscape for Idiopathic Pulmonary Fibrosis. Drugs.

[106] Vancheri C., Kreuter M., Richeldi L., Ryerson C.J., Valeyre D., Grutters J.C., Wiebe S., Stansen W., Quaresma M., Stowasser S. (2018). Nintedanib with Add-on Pirfenidone in Idiopathic Pulmonary Fibrosis: Results of the INJOURNEY Trial. Am. J. Respir. Crit. Care Med..

[107] Flaherty K.R., Fell C.D., Huggins J.T., Nunes H., Sussman R., Valenzuela C., Petzinger U., Stauffer J.L., Gilberg F., Bengus M. (2018). Safety of Nintedanib Added to Pirfenidone Treatment for Idiopathic Pulmonary Fibrosis. Eur. Respir. J..

[108] Dong Q., Bao H., Wang J., Shi W., Zou X., Sheng J., Gao J., Guan C., Xia H., Li J. (2023). Liver Fibrosis and MAFLD: The Exploration of Multi-Drug Combination Therapy Strategies. Front. Med..

[109] Pockros P.J., Fuchs M., Freilich B., Schiff E., Kohli A., Lawitz E.J., Hellstern P.A., Owens-Grillo J., Van Biene C., Shringarpure R. (2019). CONTROL: A Randomized Phase 2 Study of Obeticholic Acid and Atorvastatin on Lipoproteins in Nonalcoholic Steatohepatitis Patients. Liver Int..

[110] Muñoz-Félix J.M., González-Núñez M., Martínez-Salgado C., López-Novoa J.M. (2015). TGF-β/BMP Proteins as Therapeutic Targets in Renal Fibrosis. Where Have We Arrived after 25 Years of Trials and Tribulations?. Pharmacol. Ther..

[111] Zhang Y., Pan Z. (2025). Ovarian Fibrosis: Mechanistic Insights and Emerging Therapeutic Horizons. Gene.

[112] Lv K., Wang Y., Lou P., Liu S., Zhou P., Yang L., Lu Y., Cheng J., Liu J. (2022). Extracellular Vesicles as Advanced Therapeutics for the Resolution of Organ Fibrosis: Current Progress and Future Perspectives. Front. Immunol..

[113] Ansari Z., Chaurasia A., Neha, Sharma N., Bachheti R.K., Gupta P.C. (2025). Exploring Inflammatory and Fibrotic Mechanisms Driving Diabetic Nephropathy Progression. Cytokine Growth Factor. Rev..

[114] Macias-Ceja D.C., Mendoza-Ballesteros M.T., Ortega-Albiach M., Barrachina M.D., Ortiz-Masià D. (2023). Role of the Epithelial Barrier in Intestinal Fibrosis Associated with Inflammatory Bowel Disease: Relevance of the Epithelial-to Mesenchymal Transition. Front. Cell Dev. Biol..

[115] Huang X., Song Y., Wei L., Guo J., Xu W., Li M. (2023). The Emerging Roles of Ferroptosis in Organ Fibrosis and Its Potential Therapeutic Effect. Int. Immunopharmacol..

[116] Ning Z.-H., Wang X.-H., Zhao Y., Ou Y., Yang J.-Y., Tang H.-F., Hu H.-J. (2025). Ferroptosis in Organ Fibrosis: Mechanisms and Therapeutic Approaches. Int. Immunopharmacol..

[117] Zhang G., Wu K., Jiang X., Gao Y., Ding D., Wang H., Yu C., Wang X., Jia N., Zhu L. (2024). The Role of Ferroptosis-Related Non-Coding RNA in Liver Fibrosis. Front. Cell Dev. Biol..

[118] Dong L., Lin L.-C., Mao S., Liu Z.-Y., Liu P., Li R., Tao H., Zhang Y. (2025). Piezo-Mediated Mechanically Activated Currents in Organ Fibrosis: Novel Mechanisms and Therapeutic Strategies. Life Sci..

[119] Mikami Y., Takada Y., Hagihara Y., Kanai T. (2018). Innate Lymphoid Cells in Organ Fibrosis. Cytokine Growth Factor. Rev..

[120] Jiao M., Zhang Y., Song X., Xu B. (2024). The Role and Mechanism of TXNDC5 in Disease Progression. Front. Immunol..

[121] Bao Y.-N., Yang Q., Shen X.-L., Yu W.-K., Zhou L., Zhu Q.-R., Shan Q.-Y., Wang Z.-C., Cao G. (2024). Targeting Tumor Suppressor P53 for Organ Fibrosis Therapy. Cell Death Dis..

[122] Ramachandran P., Henderson N.C. (2016). Antifibrotics in Chronic Liver Disease: Tractable Targets and Translational Challenges. Lancet Gastroenterol. Hepatol..

[123] Li J., Dang Y., Liu M., Gao L., Lin H. (2025). Fibroblasts in Heterotopic Ossification: Mechanisms and Therapeutic Targets. Int. J. Biol. Sci..

[124] De Blasio M.J., Ohlstein E.H., Ritchie R.H. (2023). Therapeutic Targets of Fibrosis: Translational Advances and Current Challenges. Br. J. Pharmacol..

[125] Yang H., Cheng H., Dai R., Shang L., Zhang X., Wen H. (2023). Macrophage Polarization in Tissue Fibrosis. PeerJ.

[126] Hu W., Ma Z., Jiang S., Fan C., Deng C., Yan X., Di S., Lv J., Reiter R.J., Yang Y. (2016). Melatonin: The Dawning of a Treatment for Fibrosis?. J. Pineal Res..

[127] Tacke F., Zimmermann H.W. (2014). Macrophage Heterogeneity in Liver Injury and Fibrosis. J. Hepatol..

